# Effect of C282Y Genotype on Self-Reported Musculoskeletal Complications in Hereditary Hemochromatosis

**DOI:** 10.1371/journal.pone.0122817

**Published:** 2015-03-30

**Authors:** António Camacho, Thomas Funck-Brentano, Márcio Simão, Leonor Cancela, Sébastien Ottaviani, Martine Cohen-Solal, Pascal Richette

**Affiliations:** 1 Department of Orthopedics, Centro Hospitalar Lisboa Central, Lisboa, Portugal; 2 Department of Biomedical Sciences and Medicine and Centre of Marine Sciences, University of Algarve, Faro, Portugal; 3 Université Paris Diderot, Assistance Publique-Hôpitaux de Paris, Hôpital Bichat, Service de Rhumatologie, Paris, France; 4 Institut National de la Santé et de la Recherche Médicale U1132, Hôpital Lariboisière, Paris, France; 5 Université Paris Diderot, Assistance Publique-Hôpitaux de Paris, Hôpital Lariboisière, Fédération de Rhumatologie, Paris Cedex 10, France; RWTH Aachen, GERMANY

## Abstract

**Objective:**

Arthropathy that mimics osteoarthritis (OA) and osteoporosis (OP) is considered a complication of hereditary hemochromatosis (HH). We have limited data comparing OA and OP prevalence among HH patients with different hemochromatosis type 1 (HFE) genotypes. We investigated the prevalence of OA and OP in patients with HH by C282Y homozygosity and compound heterozygosity (C282Y/H63D) genotype.

**Methods:**

A total of 306 patients with HH completed a questionnaire. Clinical and demographic characteristics and presence of OA, OP and related complications were compared by genotype, adjusting for age, sex, body mass index (BMI), current smoking and menopausal status.

**Results:**

In total, 266 of the 306 patients (87%) were homozygous for C282Y, and 40 (13%) were compound heterozygous. The 2 groups did not differ by median age [60 (interquartile range [IQR] 53 to 68) vs. 61 (55 to 67) years, P=0.8], sex (female: 48.8% vs. 37.5%, P=0.18) or current smoking habits (12.4% vs. 10%, P=0.3). As compared with compound heterozygous patients, C282Y homozygous patients had higher median serum ferritin concentration at diagnosis [1090 (IQR 610 to 2210) vs. 603 (362 to 950) µg/L, P<0.001], higher median transferrin saturation [80% (IQR 66 to 91%) vs. 63% (55 to 72%), P<0.001]) and lower median BMI [24.8 (22.1 to 26.9) vs. 26.2 (23.5 to 30.3) kg/m2, P<0.003]. The overall prevalence of self-reported OA was significantly higher with C282Y homozygosity than compound heterozygosity (53.4% vs. 32.5%; adjusted odds ratio [aOR] 2.4 [95% confidence interval 1.2–5.0]), as was self-reported OP (25.6% vs. 7.5%; aOR 3.5 [1.1–12.1]).

**Conclusion:**

Patients with C282Y homozygosity may be at increased risk of musculoskeletal complications of HH.

## Introduction

Hereditary hemochromatosis (HH) is an autosomal recessive disorder characterized by increased absorption of dietary iron and rapid iron release from macrophages, which leads to abnormal accumulation of iron in several organs, particularly the liver, joints and bones [1, 2]. In patients with iron overload, 2 hemochromatosis type 1 (HFE) genotypes have been commonly described: C282Y homozygosity and C282Y/H63D compound heterozygosity. Among patients of northern European descent, the most common genotype is C282Y homozygosity, accounting for about 80% to 90% of HH cases [2].

The musculoskeletal complications of HH have been mainly described in C282Y homozygous patients. These complications consist of an arthropathy that mimics osteoarthritis (OA) and osteoporosis (OP) [3, 4]. The prevalence of OA ranges from 20% to 80% in homozygous patients with HH [4]. In addition, such patients are at increased risk of joint replacement [5]. The reported prevalence of OP in these patients ranges from 25% to 35% and OP seems to be associated with severity of iron overload [1].

Recently, there has been renewed interest in the role of HFE compound heterozygosity (C282Y/H63D) in the development of HH. Patients with the C282Y/H63D genotype show iron overload but to a lesser extent than in homozygotes, particularly in the absence of co-morbid factors [6, 7]. Little is known about the risk of hemochromatosis-related musculoskeletal complications with HFE compound heterozygosity.

We aimed to compare the prevalence of HH-related OA and OP in patients with C282Y homozygosity and those with compound heterozygosity (C282Y/H63D).

## Patients and Methods

### Patients with HH

Patients who were members of the Association Hémochromatose France (AHF) completed a self-administered questionnaire that was previously described [8]. Patients were informed of the purpose of the study and gave their informed consent to be in the study. This study was conducted in accordance with the recommendations of the Helsinki Declaration. The Institutional Review Board (IRB00006477- Comité d’évaluation de l’éthique des projets de recherche biomédicale du GHU Nord-Hôpital Bichat, Paris) reviewed and approved the study (no. 10–074).

We collected the following data: demographic characteristics (age, sex, weight, height, current smoking habit, menopausal status); details of HH history (HFE genotype, ferritin concentration and transferrin saturation at diagnosis, symptoms before diagnosis); general clinical features (asthenia, diabetes mellitus, heart disease); joint and spine involvement (patients were asked if they had received a diagnosis of OA by a physician; if they had ever complained of low back pain or sciatica; if they had knee-, hip-, or ankle-replacement prosthesis); and bone involvement (patients were asked if they had received a diagnosis of OP by a physician; if they had a history of fractures). To confirm the validity of the genotype reported by patients, we reviewed the medical records of a sample of 20 patients followed in our department who had reported having homozygosity (n = 10) and duplex heterozygosity (n = 10). The rate of concordance was 95% (19 of 20). One patient who declared homozygosity was actually compound heterozygous.

### Statistical analysis

Continuous variables were tested for normality and homoscedasticity. Some continuous variables were non-normally distributed (skewed). Therefore, variables were described with the median and interquartile range (IQR). Differences between groups were assessed by the Mann-Whitney test. The association between categorical variables was tested by chi-square test. Crude odds ratios (ORs) were calculated by an univariate logistic regression model, with the patient genotype (C282Y/C282Y vs. C282Y/H63D) as the independent variable and the outcome of interest as the dependent variable. Adjusted ORs (aORs) were calculated by a multiple logistic regression model, with the patient genotype and clinically relevant confounders as independent variables and the outcome of interest as the dependent variable. The design of the study precluded the assessment of iron overload by liver biopsy, so we used ferritin concentration as a surrogate marker, with severity of iron overload defined by serum ferritin concentration ≥1000 μg/L at diagnosis. This cut-off was previously found clinically appropriate [9]. All statistical analyses involved use of Stata 13.1 (StataCorp, College Station, TX). A 2-tailed p <0.05 was considered statistically significant.

## Results

### Demographic and clinical characteristics of patients

In total, 306 patients with HH completed the questionnaire. The sample characteristics are shown in Table 1. The median age of patients was 60 years (IRQ 53 to 68 years); 47.4% were females (80% menopausal). Overall, 266 patients (87%) were C282Y/C282Y homozygous and 40 (13%) were C282Y/H63D compound heterozygous. Median serum ferritin concentration at diagnosis was significantly higher with C282Y homozygosity than compound heterozygosity [1090 (IQR 610 to 2210) vs. 603 (362 to 950) μg/L, P<0.001], as was median transferrin saturation [80% (IQR 66 to 91%) vs. 63% (55 to 72%), P<0.001]. The proportion of patients with severe iron overload (> 1000 μg/L) was greater with C282Y homozygosity than C282Y/H63D compound heterozygosity (52.4% vs. 20%, P< 0.001).

**Table 1:** Characteristics of patients with hereditary hemochromatosis by genotype.

	C282Y/C282Y n = 266	C282Y/H63D n = 40	P value
Age, years	60 (53–68)	61 (55–67)	0.50
Women, %	48.8	37.5	0.18
Body mass index, kg/m^2^	24.8 (22.1–26.9)	26.2 (23.5–30.3)	0.003
Menopausal women, %	77.6	80	0.80
Current smoker, %	13.2	7.5	0.30
Serum ferritin concentration at diagnosis, μg/L	1090 (610–2210)	603 (362–950)	< 0.001
Transferrin saturation, %	80 (66–91)	63 (55–72)	< 0.001

Data are median (interquartile range) or percentage. NS: not significant

### Self-reported symptoms at the time of the survey

The prevalence of self-reported joint pain at the time of the survey was significantly higher with C282Y homozygosity than C282Y/H63D compound heterozygosity (88.4% vs. 77.5%, aOR = 2.8 [95% confidence interval (95% CI) 1.2–6.9]; Table 2). The prevalence of self-reported asthenia, diabetes and cardiac disease did not differ between the genotypes.

**Table 2:** Prevalence of self-reported symptoms at the time of the survey by genotype.

	C282Y/C282Y n = 266	C282Y/H63D n = 40	Crude OR (95% CI)	aOR*(95% CI)	P value
Joint pain, %	88.4	77.5	2.2 (0.96–5.1)	2.8 (1.2–6.9)	0.02
Asthenia, %	79.3	82.1	0.82 (0.34–1.9)	0.8 (0.33–1.9)	0.56
Diabetes, %	9.7	10	0.98 (0.32–2.9)	1.2 (0.37–3.9)	0.87
Cardiac disease, %	15.4	17.5	0.86 (0.36–2.1)	0.98 (0.39–2.5)	0.83

OR, odds ratio; aOR, adjusted OR; 95% CI, 95% confidence interval.

*Adjusted for age, sex, body mass index and current smoking habits.

### OA, joint replacement and spine involvement

The prevalence of OA was higher with C282Y homozygosity than C282Y/H63D compound heterozygosity: 53.4% vs. 32.5% (aOR = 2.4 [95% CI 1.2–5.0]; Table 3). This difference was no longer significant on adjustment for ferritin concentration (aOR = 1.66 [0.79–3.5]). The prevalence of hip replacement was higher for homozygotes than heterozygotes--11.7% vs. 7.5% (aOR = 1.5 [0.41–5.1])—but this difference did not reach statistical significance. The prevalence of self-reported back pain and sciatica did not differ between the groups: 71.8% vs. 70% and 47.4% vs. 45%, respectively.

**Table 3:** Prevalence of osteoarthritis, joint replacement, back pain and sciatica by genotype.

	C282Y/C282Y n = 266	C282Y/H63D n = 40	Crude OR(95% CI)	aOR*(95% CI)	P value
Osteoarthritis, %	53.4	32.5	2.4 (1.2–4.8)	2.4 (1.2–5)	0.02
Knee replacement, %	5.6	5.0	1.1 (0.25–5.1)	1.5 (0.3–7.1)	0.64
Hip replacement, %	11.7	7.5	1.6 (0.47–5.6)	1.5 (0.41–5.1)	0.08
Ankle replacement, %	0.75	2.5	0.3 (0.03–3.3)	0.32 (0.025–4)	0.52
Back pain, %	71.8	70	1.1 (0.53–2.3)	1.2 (0.55–2.6)	0.31
Sciatica, %	47.4	45	1.1 (0.56–2.1)	1.3 (0.65–2.6)	0.59

* Adjusted for age, sex and body mass index.

### OP and fractures

In total, 155 patients (50.7%) reported than they had undergone dual-energy X-ray absorptiometry (DEXA). All the patients who self-reported OP also reported having undergone DEXA. The prevalence of OP was greater for C282Y homozygous than compound heterozygous patients (25.6% vs. 7.5%; aOR = 3.5 [95% CI 1.1–12.1]); Table 4). The effect of genotype on the prevalence of OP was no longer statistically significant on adjustment for ferritin concentration (aOR = 2.6 [0.74–9.3]). The prevalence of hip, wrist and vertebral fractures did not differ between the groups: 2.6% vs. 2.5%, 13.2% vs. 7.5% and 7.9% vs. 2.5%, respectively.

**Table 4:** Prevalence of osteoporosis and associated fractures by genotype

	C282Y/C282Y n = 266	C282Y/H63D n = 40	Crude OR(95% CI)	aOR*(95% CI)	P value
Osteoporosis, %	25.6	7.5	4.3 (1.3–14.2)	3.5 (1.1–12.1)	0.04
Hip fracture, %	2.6	2.5	1.1(0.13–8.8)	0.66 (0.07–6.3)	0.64
Wrist fracture, %	13.2	7.5	1.9 (0.55–6.4)	2.3 (0.65–8.2)	0.45
Vertebral fracture, %	7.9	2.5	3.3 (0.44–25.6)	3.3 (0.42–26)	0.15

* Adjusted for age, sex, body mass index, menopausal status and smoking habits

## Discussion

We investigated the prevalence of OA and OP, HH-related musculoskeletal complications, by C282Y homozygosity and compound heterozygosity (C282Y/H63D) genotype and found the prevalence of both self-reported OA and OP higher for patients with C282Y homozygosity than compound heterozygosity.

Our study population consisted of 306 patients, the C282Y/C282Y genotype being 6.7 times more frequent than the C282Y/H63D genotype, which agrees with previous studies of patients with HH-related musculoskeletal complications [10, 11]. As expected, as compared with patients with compound heterozygosity, those with the homozygous C282Y/C282Y genotype showed significantly higher ferritin concentration and transferrin saturation values, and about half of the homozygous patients showed severe iron overload. BMI was greater for compound heterozygous than homozygous patients, as was found recently [6]. Indeed, co-morbid factors such as alcohol abuse and overweight are more prevalent in C282Y/H63D than C282Y/C282Y patients [6].

In HH, abnormally increased iron absorption leads to iron loading of parenchymal cells in the liver, pancreas, heart and joints, with subsequent damage to structure and impaired function [2]. The reasons for the high frequency of the musculoskeletal system involvement in HH are unknown [2].

In this study, the 2 genotype groups did not differ in prevalence of self-reported asthenia, cardiac disease or diabetes, which is similar to findings by Waalen et al. [12], showing that patients with different genotypes of HFE self-reported similar rates of symptoms commonly associated with iron overload.

Several studies have well documented the association of HH and an arthropathy that may seriously affect quality of life [1, 8, 13]. This arthropathy can be severe, as highlighted by the high prevalence of hip, knee and ankle joint replacement in these patients [5, 8, 10].

In our study, the prevalence of self-reported OA was greater in patients with C282Y homozygosity than C282Y/H63D compound heterozygosity, after adjustment for confounding variables. The prevalence of hip replacement surgery was greater but not significantly for C282Y homozygous than compound heterozygous patients, as was found recently [14]. This increased prevalence of OA in C282Y homozygotes might be related to the more severe iron overload in these patients. Indeed, this difference was no longer significant after adjustment for ferritin concentration. Although the role of iron overload in the genesis of HH-related OA is still not fully understood, iron overload can lead to iron accumulation in the synovium and cartilage of HH patients, as seen on histology [1], and to increased ferritin concentration in synovial fluid [15]. Some of this iron can be in the form of non-transferrin–bound iron and might have deleterious effects [16] on synovial tissue, promoting inflammation with neutrophil infiltration [17] and tissue damage, as was demonstrated in the liver [18]. Iron deposits were found in synovial tissue from patients with inflammatory arthritis [1], which suggests a link between iron content and inflammation. More recently, the serum level of vascular adhesion molecule 1 was found associated with radiographic measures of HH arthropathy, which suggests its involvement in HH joint damage [19].

Patients with HH have shown disc degeneration and calcifications in the intervertebral discs due to calcium pyrophosphate crystal deposits [20]. In a retrospective study [21], one third of patients with HH showed spine arthropathy on radiography. The literature contains no data on the prevalence of low back pain in patients with a clinical phenotype of HH. Both our genotype groups showed similar self-reported prevalence of low back pain and sciatica, which suggests that the lumbar spine involvement in HH is independent of the genotype.

We found an increased prevalence of OP, along with increased prevalence, although not significant, of vertebral fractures in homozygote patients. Again, this observation could be related to the increased iron overload observed in C282Y homozygotes. Indeed, the impact of the genotype on the prevalence of OP was no longer significant when we adjusted for ferritin concentration. Several reports have shown that HH patients, particularly males, are at increased risk of OP, which might be related to iron overload [1, 22].

The pathophysiology of OP in HH is not entirely clear. Animal and *in vitro* data support that iron excess can affect bone formation and remodeling [1]. Iron-overloaded mice show increased level of reactive oxygen species, serum tumor necrosis factor α and interleukin 6, associated with severity of iron overload [23]. HFE-/- mice show impaired bone microarchitecture and increased osteoclast number [24]. *In vitro*, iron disrupts the formation of hydroxyapatite crystals and inhibits the proliferation and differentiation of osteoblasts [1].

There are several limitations and caveats to this study. Patients were asked to remember symptoms or fractures attributed to hemochromatosis, which might suggest recall bias. As for all surveys, this study may lack robust internal validity. Because of the relatively low number of patients with compound heterozygosity, the number of rare events such as fractures or joint replacement surgery was low in this group, which might explain the non-significant findings for joint replacement and fracture. Finally, we could not differentiate symptomatic and asymptomatic fractures.

## Conclusions

To conclude, this is the first study to compare the effect of genotype on HH-related musculoskeletal symptoms. C282Y homozygosity may confer increased risk of OA and OP as compared with C282Y/H63D compound heterozygosity.
